# The ability of *Phaeobacter inhibens* to produce tropodithietic acid influences the community dynamics of a microalgal microbiome

**DOI:** 10.1038/s43705-022-00193-6

**Published:** 2022-11-03

**Authors:** Nathalie Nina Suhr Eiris Henriksen, Morten Dencker Schostag, Simone Rosen Balder, Pernille Kjersgaard Bech, Mikael Lenz Strube, Eva Christina Sonnenschein, Lone Gram

**Affiliations:** 1 Department of Biotechnology and Biomedicine, Technical University of Denmark, Søltofts Plads Bldg. 221, DK-2800 Kgs, Lyngby, Denmark; 2 Present Address: Department of Biosciences, Swansea University, Singleton Park, SA2 8PP Swansea, United Kingdom

**Keywords:** Microbiome, Molecular evolution

## Abstract

Microbial secondary metabolites facilitate microbial interactions and are crucial for understanding the complexity of microbial community dynamics. The purpose of the present study was to determine how a secondary metabolite producing marine bacteria or its metabolite deficient mutant affected the microbiome of the marine microalgae *Tetraselmis suecica* during a 70 day long co-evolution experiment. Using 16S rRNA gene amplicon sequencing, we found that neither the tropodithietic acid (TDA)-producing *Phaeobacter inhibens* wildtype nor the TDA-deficient mutant had major impacts on the community composition. However, a subset of strains, displayed temporally different relative abundance trajectories depending on the presence of *P. inhibens*. In particular, a *Winogradskyella* strain displayed temporal higher relative abundance when the TDA-producing wildtype was present. Numbers of the TDA-producing wildtype were reduced significantly more than those of the mutant over time indicating that TDA production was not an advantage. In communities without the *P. inhibens* wildtype strain, an indigenous population of *Phaeobacter* increased over time, indicating that indigenous *Phaeobacter* populations cannot co-exist with the TDA-producing wildtype. Despite that TDA was not detected chemically, we detected transcripts of the *tdaC* gene indicating that TDA could be produced in the microbial community associated with the algae. Our work highlights the importance of deciphering longitudinal strain dynamics when addressing the ecological effect of secondary metabolites in a relevant natural community.

## Introduction

Bacteria evolve in complex and interacting microbial communities, where their geno- and phenotypes are shaped by interspecies interactions that can be facilitated by spatial organization, metabolic dependencies, or the production of secondary metabolites. Microbial secondary metabolites may play important ecological roles in microbial communities, ranging from all-out chemical conflict [1] to cooperation mediators [2]. Untangling the effect of secondary metabolites in natural microbial communities requires targeted manipulations, and consequently, interspecies interactions have often been explored in simple systems consisting of a few species [3, 4]. The addition of more species may alter the outcomes considerably [5] and there is a need for microbial model systems with intermediate complexity to assess the ecological roles of microbial secondary metabolites [4]. In the present study, we focus on a marine algal system, in which members of the *Roseobacter* group are naturally present and of which some are potent producers of secondary metabolites [6].

The paraphyletic *Roseobacter* group represents one of the most abundant and omnipresent groups of marine bacteria [7, 8], constituting 2–8% of microbial communities in surface waters [9]. The abundance of this group is positively correlated to chlorophyll *a* and can constitute up to 40% of the microbial communities during coastal algal blooms [9, 10], signifying its association with microalgae. Especially clade 1 roseobacters are prominent producers of secondary metabolites, such as indigoidine [11], and tropodithietic acid (TDA) [12–14]. The species *Phaeobacter inhibens* is a member of clade 1 and is often found in microbial communities associated with micro- and macroalgae [15, 16] and it has been suggested that the secondary metabolites produced by *Phaeobacter* species can control pathogenic microorganisms and other community members [17, 18], pointing to *P. inhibens* as a strong modulator of the host microbiome.

TDA is a broad-spectrum antimicrobial secondary metabolite affecting a wide range of both Gram-positive and Gram-negative bacteria [13, 19–21], with a suggested mode of action as a proton antiporter at the cytoplasmic membrane [22], explaining why resistance towards TDA does not arise easily [19, 23]. At sub-lethal concentrations, TDA may act as a signaling molecule affecting motility, biofilm formation and self-induction of secondary metabolite production in TDA-producers [24]. However, the concentrations, at which TDA is produced in natural communities are unknown [17], and, hence it is not known if the compound acts primarily as a signal or as an antimicrobial in natural niches.

TDA or TDA-producing *P. inhibens* can affect the taxonomic composition in several eukaryotic-host associated microbiomes [6, 18, 25, 26]. Closely related taxa, such as bacteria belonging to the *Rhodobacteraceae* family, decrease in relative abundance in the presence of *P. inhibens* or pure TDA [6, 18, 25, 26], and bacteria such as *Vibrio* and *Pseudoalteromonas* species are diminished in the presence of *P. inhibens* [18, 25]. Nevertheless, these microbiome studies have been conducted on a short-term time scale, examining the influence of TDA-producing *P. inhibens* at a maximum period of 8 days; however, microbial communities can go through distinct stages of community succession [27–30], in which the response of the community to e.g., perturbations can depend on the present state and complexity of the community [6].

**Fig. 1:**
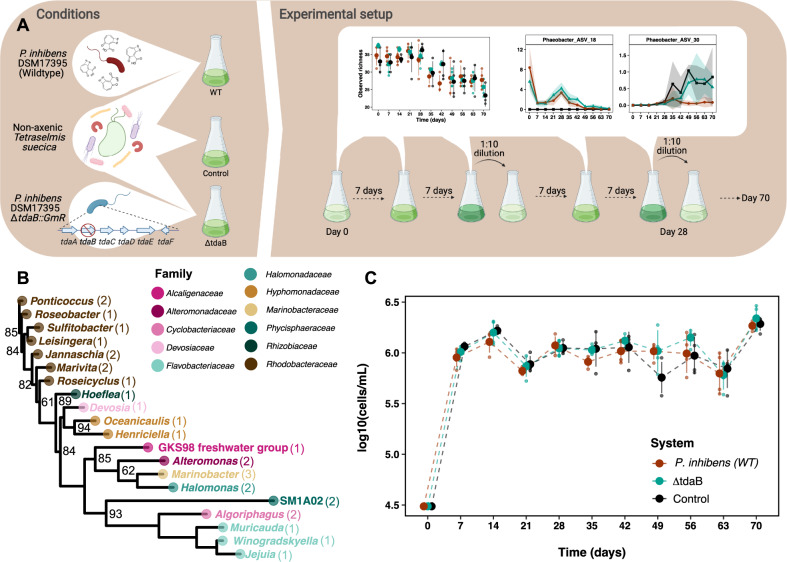
The microalgae represent a stable and healthy host-community over two months of exposure to *Phaeobacter inhibens*. **A** Illustration of our experimental protocol. Three different systems (conditions) were established consisting of non-axenic *Tetraselmis suecica* cultures subjected to either *Phaeobacter inhibens* DSM17395 (WT), *P. inhibens* DSM17395 *tdaB::GmR* (ΔtdaB), or no exposure (Control). Four biological replicates (lineages) were established for each system (*N* = 4). Samples were taken every seventh day, and at every 14th day a fraction of each lineage was tenfold diluted in fresh medium. This cycle was repeated until day 70. Illustration created with Biorender.com. **B** Phylogenetic tree of the top dominant taxa across all three systems, with identified genus names labeled and colored by family. Brackets indicate the number of detected ASVs belonging to each genus. Numbers in black represent bootstrap values. **C** Abundance of *T. suecica* over time, colored according to experimental condition. The abundance was similar between all three systems at all timepoints (*p* > 0.05, LMM). Data is based on four biological replicates (*N* = 4).

The purpose of the present study was, on a longer time scale, to investigate the influence of TDA-producing *P. inhibens* on the bacterial community associated with the marine microalgae *Tetraselmis suecica*. Addressing specifically the effect of the ability to produce TDA, we also included a TDA-negative *P. inhibens* variant (Δ*tdaB::GmR*) [31]. We followed the microbial community dynamics of the marine green alga *T. suecica’s* bacterial communities, exposed to the wildtype TDA-producer or TDA-deficient mutant, for a total period of 70 days (Fig. 1A). We transferred and propagated communities at a low dilution factor (1:10) every 14 days, to maintain high diversity and allow for continuous algal and microbial growth.

Our study has a major advantage as experimental propagation over time of the communities allowed us to shield them from external perturbations and any observed community dynamics were hence a result of intrinsic processes. Also, the microalgal host acts as the only primary carbon provider. Thus, the system mimics the ecological conditions, from which we derived the microbial community, and hence a natural niche for a TDA-producer.

## Materials and methods

### Algal and bacterial strains

Non-axenic *T. suecica* was provided by a commercial aquaculture facility and grown in a sterilized modified version of Guillard’s f/2 medium [32], without Na_2_SiO_3_ and with 5 mM NH_4_Cl in 3% Instant Ocean® Sea Salt (Aquarium Systems Inc., Sarrebourg, France). Cultures were grown stagnant, at 18 °C with white fluorescent light (24.31 µmol m^−2^ s^−1^). *Phaeobacter inhibens* DSM17395 [20, 33] and the TDA-deficient transposon mutant (*tdaB::GmR*) [31] was used as ancestral strains. Strains from freeze stocks were streaked on Marine Agar (MA; 2216 Difco) and inoculated in Marine Broth (MB; 2216 Difco) with agitation (200 rpm). Both agar and liquid cultures were incubated at 25 °C. One colony was used per biological replicate.

### Longitudinal experimental setup

Exponentially growing *T. suecica* was re-inoculated in f/2 medium to log 4.5 ± 0.12 cells mL^−1^ and divided into 12 cultures of 150 mL in 1 L Erlenmeyer flasks. The cell density of *T. suecica* was determined using an improved Neubauer counting chamber. In quadruplicates, exponential growing cultures of *P. inhibens* and the TDA-deficient mutant in MB were washed once in f/2 medium (3000 × *g*, 3 min), and adjusted to approximately log 6.2 CFU mL^−1^ using optical density (OD_600nm_). *P. inhibens* and the TDA-deficient mutant were each inoculated in four *T. suecica* cultures at a final concentration of approximately log 4 CFU mL^−1^. Inoculation levels were confirmed by CFU counts on MA. The remaining four *T. suecica* cultures served as controls and sterile f/2 was added in a volume like the cultures with bacterial inoculum.

The evolution experiment was conducted as serial batch transfers, where every other week the content of each culture was homogenized by swirling and 15 mL was transferred to a new 1 L Erlenmeyer flask, containing 135 mL f/2 medium, resulting in a 1:10 dilution (Fig. 1A). Every week, before transfer, the cultures were sampled for DNA, bacterial isolation of *P. inhibens* and algal abundance. For algal abundance measures, 1 mL of each culture was fixed in a final concentration of 1% 0.2 µm-filtered glutaraldehyde, and algal cells counted using an improved Neubauer counting chamber. For bacterial isolation of *P. inhibens*, serial dilutions were plated on MA and colonies were isolated after 72 h at 25 °C. For DNA extraction, 50 mL of each culture was pelleted (8000 × *g*, 5 min, 25 °C) and resuspended in 1 mL lysis buffer (400 mM sodium chloride, 750 mM sucrose, 20 mM EDTA, 50 mM Tris-HCl, 1 mg mL^−1^ lysozyme, pH 8.5) [34] and stored at −80 °C until extraction.

### DNA extraction and amplicon sequencing of the 16S rRNA V3V4 region

DNA extractions were performed using the phenol/chloroform-based protocol [18]. DNA was eluted in Tris-HCL buffer (pH 8.5) and incubated at 4 °C overnight. Concentrations and quality of the DNA was determined by fluorescence (Qubit^TM^ dsDNA HS assay; Invitrogen by Thermo Fisher Scientific Inc., Eugene, OR, USA) spectroscopy and absorption (DeNovix 439 DS-11+, DeNovix Inc., Wilmington, DE, USA), respectively. Prior to amplification, DNA samples were diluted to the same concentration (15 ng µL^−1^) for all samples, except samples with lower DNA yield, which were used undiluted. The DNA was used for PCR amplification of the 16S rRNA gene V3V4 region using primers tagged with octameric barcodes. For each PCR reaction, 10.6 µL DNase-free water, 12.5 µL TEMPase Hot Start 2 × Master Mix Blue II (Ampliqon, 290806), 0.8 µL of each forward primer (Fw_341; 5’CCTACGGGNGGCWGCAG’3) and reverse (Rv_804; 5’GACTACHVGGGTATCTAATCC’3) [35] primer (stock conc. 10 µM) and 0.3 µl DNA template were used. The PCR amplification program consisted of 95 °C for 15 min, followed by 30 cycles, with 1 cycle consisting of 95 °C for 30 s, 62 °C for 30 s, and 72 °C for 30 s, and a final step of 72 °C for 5 min. The PCR products were purified using Ampure XP magnetic beads purification (0.6:1 bead volume to DNA solution; Agencourt Bioscience Corporation, Beverly, MA, USA). The PCR products’ concentration and quality were assessed as described above and pooled in equimolar ratios. Amplicons were sent to Novogene (Cambridge, United Kingdom) for 250PE sequencing on an Illumina Novaseq 6000 platform.

### Enumeration of *P. inhibens* by quantitative PCR in the experimental evolution setup

Total *P. inhibens* abundance was estimated using qPCR with species specific primers [18]. Standard curves based on DNA from dilution series of *P. inhibens* DSM17395 were used to relate the threshold cycle (C_T_-value) to CFU mL^−1^. For each qPCR reaction, PowerUp™ SYBR® Green 2 x Master Mix (ThermoFischer, A25742) and 0.7 µM (final concentration) of each forward (Pi_Fw; 5’GTG TGT TGC GGT CTT TCA CC’3) and reverse primer (Pi_Rev; 5’AGG ACC ATG TCC CCT CTA CC’3) were used with 1 µL of DNA template from each dilution in duplicates. No Template Controls (NTCs) were included containing sterile water. The final reaction volume was 15 µL for all reactions. A 2-step PCR amplification, followed by a melting curve, was performed with the thermal cycler MX3000P instrument (Stratagene, La Jollla, CA). SYBR Green was detected as the fluorescent tag, while ROX acted as the reference dye. The annealing/elongation temperature was 60 °C. All samples were run in three technical replicates.

### Quantification of *tda*C transcripts in the microalgae community

The level of expression of *tdaC*, a biosynthetic gene of *Phaeobacter* TDA synthesis, was determined in a microalgal experimental setup identical to the longitudinal experimental setup with sampling at day zero, one, four, and seven. Preparation of *P. inhibens*, non-axenic *T. suecica* and abundance calculations were conducted as described in the experimental evolution setup above, but with a starting volume of 300 mL. For RNA and DNA extraction, 45 mL of each culture (*N* = 4) was sampled, and 5 mL ice-cold RNA stop solution (5% water-saturated phenol in absolute ethanol [36]) was added immediately. Samples were mixed well and pelleted (12,000 × *g*, 5 min, 20 °C) followed by removal of supernatant, except for 1 mL used to resuspend the pellet and transfer the sample to a 2 mL tube. Samples were again pelleted (12,000 × *g*, 5 min, 4 °C), followed by removal of supernatant and flash freezing in liquid nitrogen. Samples were stored at −80 °C until extraction. Total RNA and DNA was isolated using the RNeasy PowerPlant Kit according to the manufacturer’s instructions, with the modification of using FastPrep (FastPrep FP120; MP Biomedicals, California, USA) for bead beating step at speed 5.5 for 30 s. Samples were eluted in 100 µl and split into 20 µl aliquots, of which one was used as a DNA sample. DNA was removed from RNA with Turbo^TM^ DNase kit (Thermo Fisher Scientific), and RNA was reverse transcribed into cDNA using SuperScript IV (Invitrogen) with *tdaC* specific reverse primer (see below). Prior to reverse transcription, samples from day four and seven were 2x diluted in molecular grade water. Primers targeting the *tdaB* and *tdaC* genes in the TDA gene cluster were designed based on *P. inhibens* DSM17395 genome (GenBank assembly accession GCA_000154765.2) using CLC main workbench (CLC Bio, QIAGEN, Aarhus, Denmark). The primers used were *tdaB*_forward (5´AATACGACCTTATCCCTGT´3), *tdaB*_reverse (5´ CCATAACCTCAAGTGGCA´3), *tdaC*_forward (5’GTTTTGGTTGGGGTGGTAG’3), *tdaC*_reverse (5’CAGAGCGTGGTGATACAG’3). Both primer sets were tested with end-point PCR on DNA extract from mono-culture of *P. inhibens* DSM17395, TDA-deficient transposon mutant (*tdaB::GmR*), and DNA extract from non-axenic *T. suecica*. The PCR products were verified by sequencing. Primers targeting *tdaC* transcripts were verified using cDNA from RNA extraction from cultures of non-axenic *T. suecica* (log 4.5 cells ml^−1^) co-cultured with *P. inhibens* DSM17395 (~log 4,8 cell ml^−1^) for three days with the same growth condition, RNA extraction, DNase treatment and RT treatments as described above. The PCR products were verified by sequencing. A qPCR standard curve was prepared adding aliquots from a 10-fold serial dilution of an O/N culture of *P. inhibens* DSM17395 to *T. suecica* culture with a concentration of log ~5.5 cells mL^−1^. The standard curve ranged from log 6.56 to log 3.56 *P. inhibens* DSM17395 ml^−1^.

Gene (*tdaB* and *tdaC*) and transcript (*tdaC*) abundance was quantified using RT qPCR in a CFX Opus 96 real-time machine (Bio-Rad Laboratories, Inc., Hercules, CA, USA). DNase treated samples were included as a control for DNA removal for the cDNA samples. qPCR amplification was performed in 20 μL using Luna Universal qPCR Master Mix (New England Biolabs) with the *tdaB* and *tdaC* primers as described above, in a concentration of 0.25 µM and 1 µl of either template DNA, DNase treated RNA or cDNA. A two-step cycle protocol was employed following the manufacturer’s instructions with an annealing temperature of 60 and 63 °C, for *tdaB* and *tdaC,* respectively. Water, no template (NTC) and no reverse-transcriptase controls were included as controls. Melting curves from all analyses were examined for verification of the right amplification product. The efficiency for *tdaB* amplification was 97.0% and the R^2^ value was 0.998. For *tdaC* gene and transcript detection, the efficiency ranged from 94.7–99.9% and R^2^ values were between 0.997–0.999. Gene abundance and transcript abundance was calculated from the standard curve. We were able to detect *tdaC* transcripts down to samples containing log 5.56 *P. inhibens* ml^−1^ in the standard curve samples.

### Whole genome sequencing of evolved *P. inhibens* isolates from the experimental evolution setup

*P. inhibens* were isolated on day zero (12 isolates), 35 (12 isolates), 63 (six isolates) and 70 (18 isolates). Genomic DNA was extracted from O/N cultures using the NucleoSpin®Tissue (Macherey-Nagel, 740952.5) protocol for bacteria, with three hours of pre-lysis incubation. Quality and quantitative assessment of the extracted DNA was done as described from amplicon sequencing. Samples were sequenced by Novogene (Cambridge, United Kingdom) on the Illumina NovaSeq 6000 150PE platform.

### Whole genome assembly and bioinformatic pipeline of *P. inhibens* isolates

Reads were confirmed as being *P. inhibens* using *Centrifuge* [37] with default settings and the p + h + v database. The genomes were then *de novo* assembled using the *Shovill* pipeline (https://github.com/tseemann/shovill) using default settings. The genome assemblies were evaluated using the tool *Quast* [38] and annotated by *Prokka* [39]. Prediction of the position of the 16S rRNA gene was done using the *barrnap* tool (https://github.com/tseemann/barrnap), with a reject level at 0.7, not allowing hits with a length lower than 70% of the expected length. For Single Nucleotide Polymorphism (SNP) detection, raw reads from the 48 isolates were mapped to the reference genome of *P. inhibens* DSM17395 (GenBank assembly accession GCA_000154765.2) using the *snippy* tool (https://github.com/tseemann/snippy) with default settings.

### Bioinformatic analysis of V3V4 amplicons from the experimental evolution setup

Raw reads were demultiplexed, primers removed and read orientation corrected with *Cutadapt* 3.7 [40], using default settings. We used *DADA2* 1.16 [41] in *R* 4.0.2 [42] to denoise and join reads into exact amplicon sequence variants (ASVs). We performed denoising, joining of reads and removal of chimeras using default parameters, except the error rate for the parametric error model was trained on 10^9^ bases in total. Chimeras were removed with the consensus method and only ASVs with a length of more than 380 bp were retained. Taxonomic classification was done through the *DADA2* pipeline with the *SILVA* database release 138.1 [43] training set, using default settings, except the reverse-complement orientation of the sequences was also used for taxonomic classification. A total of 24382203 read pairs passed quality control, with a mean ± sd of 174 158.6 ± 110 439.3 reads pair per sample (Table S1). Samples with less than 5000 reads were removed. Contaminants were removed using the prevalence method with *decontam* 1.16 [44] using default settings. ASVs classified as chloroplasts and eukaryotes at phylum levels were discarded. By visual inference of the elbow point of a rank-order plot of reads per ASV across the entire dataset, only ASVs with more than 100 reads (corresponding to a relative abundance > 0.05% across the dataset) were retained in the final ASV table resulting in 46 ASVs in total.

### Downstream sequence analyses of 16S rRNA gene amplicons

All analyses were performed using R 4.2.0 [42] in RStudio [45], using the following packages: *tidyverse* [46], *ggplot2* [47], *vegan* [48], and *phyloseq* [49]. A phylogenetic tree was constructed by aligning sequences with MAFFT version 7.490 [50] using a gap penalty of 1 and adjusting sequence direction if necessary. FastTree [51] was used to create an approximately-maximum-likelihood phylogenetic tree, using generalized time-reversible (GTR). The tree was rooted by automatically picking an outgroup with the longest branch. Observed richness was calculated on the ASV table including ASVs classified as *Phaeobacter* (*phyloseq* estimate_richness). Means and standard deviations are summarized in Table S2. Identification of ASVs having differential abundance at day 42 was performed using the R package *metacoder* using default settings [52]. For beta diversity analyses, we excluded ASVs classified as *Phaeobacter* from the ASV table, prior to normalization using total sum scaling (TSS) to 100000 reads per sample. Bray–Curtis dissimilarities were calculated from the Wisconsin- and square-root transformed ASV table (*vegan* vegdist). Non-metric dimensional scaling (nMDS) ordination plots in three dimensions were used to visualize the results (*vegan* metaMDS, trymax = 1000, k = 3), since analysis of the stress-dimension plot revealed an elbow at dimension three with a stress value of 0.1 (Fig. S4). Significance of terms was determined by a PERMANOVA test (*vegan* adonis2), and homogeneity of multivariate variance was identified by beta-dispersion test (*vegan* betadisper and permutest, permutations = 1000). Multilevel pairwise comparison of the three systems was tested using pairwiseAdonis v. 0.4 [53] (*pairwiseAdonis* pairwiseadonis2). Multiple regression of ASV variables with nMDS ordination axes (*vegan* envfit, *r* > 0.5) was used to find ASVs that explained the lineage diversification in the ordination plot (Table S3). Abundance trajectories of ASVs were visualized for ASVs that were present in more than one sample. Significant different ASV profiles between the three systems was identified using linear mixed effect analysis on relative abundances (Fig. S7), as described below. Only ASVs with a significant difference between systems (*p* < 0.05, fixed effect variable: System), were included in the final analysis (Table S4).

### Mixed effects models

All statistical analyses were performed using R 4.2.0 [42] in RStudio [45]. The function *lmer* from the package *lme4* [54] was used to perform linear mixed effects analysis (LMM) of the relationship between time and system (fixed effects) and the respective dependent variable. Lineage was specified as a random effect. Each sample represented one independent observation. Inspection of studentized residuals were used to detect obvious outliers. Outliers were excluded from the analysis if the studentized residual was above 2.5. *P*-values were obtained by the *Anova* function from the car package [55] for the LMMs and post hoc multiple pairwise comparison using Estimated Marginal Means (EMMs) function *emmeans* from the package *emmeans* [56], with inclusion of *p*-value adjustment (Bonferroni).

## Results

The influence of the TDA-producer *P. inhibens* DSM 17395 (*P. inhibens* (WT)), and its TDA-deficient mutant, on the microbial community associated with the marine microalgae *T. suecica* was determined by sequencing of 16S rRNA gene V3-V4 region amplicons and comparing the taxonomic diversity, composition, and structure over 70 days (Fig. 1A). In total, 46 bacterial ASVs were observed in the microalgal microbiome across the entire time course. Out of these, 29 ASVs were distributed on 20 genera, excluding *Phaeobacter*, represented 95.1% of the total microbiome over the entire study (Fig. 1B). Of these, the top five genera were *Jejuia*, SM1A02 (family: *Phycisphaeraceae*), *Algoriphagus*, *Marivita* and *Hoeflea* with mean relative abundances of 33.1 ± 13.2%, 18.8 ± 9.0%, 9.9 ± 4.9%, 8.2 ± 8.8% and 3.7 ± 4.3%, respectively (Fig. S1).

The initial abundance of *T. suecica* was determined every seventh day and used as an indicator for system health and stability [4]. After inoculation with log 4.5 ± 0.1 cells mL^−1^, *T. suecica* abundance increased to log 6.0 ± 0.1 cells mL^−1^ during the two weeks of growth (Fig. 1C). Following the bi-weekly transfer, *T. suecica* regrew to this level, with no significant difference between the three systems (*p* = 0.11, LMM), indicating that the microalgal host was unaffected by the addition of *P. inhibens* (WT) or the TDA-deficient mutant, thus ensuring a stable model system.

### The ability of *Phaeobacter* to produce TDA is a minor driver of diversity

**Fig. 2:**
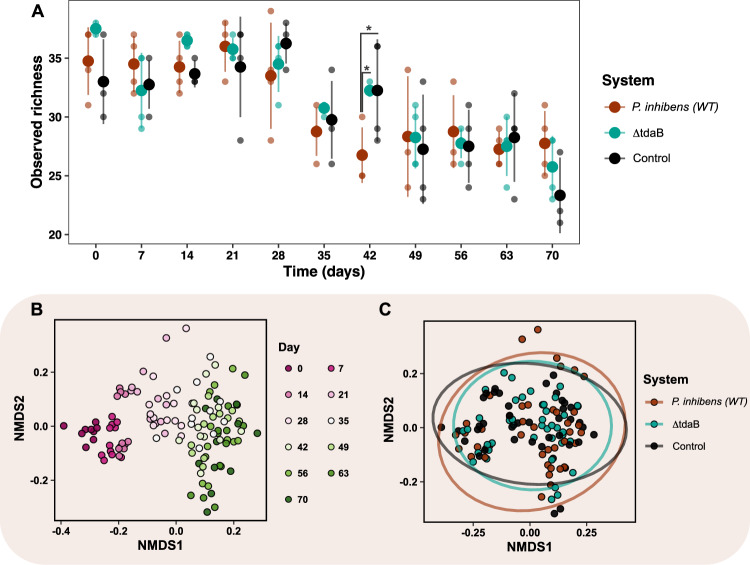
Changes in the microbial community composition and diversity. **A** Observed richness (alpha diversity depicted as number of ASVs) of the three systems in response to the addition of *Phaeobacter inhibens* DSM17395 (WT), TDA-deficient *P. inhibens* DSM17395 (ΔtdaB) and no addition (Control) over 70 days. Asterisks represent significant differences (*p* < 0.05, LMM & EMM). Filled circles represent averages and transparent points biological replicates (lineages). Error bars represent standard deviation (*N* = 4). **B** Non-metric multidimensional scaling (NMDS) plot on Bray–Curtis distances of ASV composition. The first two of three dimensions are shown. Points represent samples that are colored according to time, and **C** system. The ellipses represent 95% confidence interval. Stress = 0.09986.

If *P. inhibens* uses TDA as an antagonistic compound to kill competitors, a decrease in bacterial richness could be expected in the systems exposed to TDA-producing *P. inhibens* (WT). Therefore, the effect of *P. inhibens* strains on ASV richness of the bacterial communities was estimated as number of observed ASVs over time (Fig. 2A). No significant difference in richness was found between the three systems over time (*p* = 0.86, LMM), except from day 42, where the addition of *P. inhibens* (WT) caused a significant lower richness than found in the other two systems (*p* = 0.017, EMMs). The differences on day 42 was caused by ASVs belonging to the genera *Halomonas*, *Haliea*, *Muricauda*, *Roseobacter* and *Methylophage* not being detected in the *P. inhibens* (WT) system (Fig. S3). However, the number of observed ASVs was significantly different over time in all systems (*p* < 2e–16, LMM), explained by the observed richness decreased with 9.5 ± 2.4 ASVs from day zero to 70. The parallel reduction in richness indicates that other factors, such as the biweekly dilution of the systems, rather than the addition of the TDA-producing *P. inhibens* (WT) or a TDA-deficient mutant, drove this richness reduction over time.

### Variables influencing the microbial community composition

Although *P. inhibens* (WT) did not have strong influence on the presence/absence of ASVs in the *T. suecica* microbiome, TDA-producers could be responsible for modulating and altering the growth of community members. We used NMDS ordination to visualize Bray–Curtis dissimilarities of communities, and permutational multivariate analysis of variance (PERMANOVA) to test for significant differences. We excluded ASVs classified as *Phaeobacter* from this analysis to focus on the effect of *Phaeobacter*. The PERMANOVA revealed that 37.95% of the variation observed in the bacterial composition were explained by time and type of system (Fig. 2B, C). The bacterial communities segregated according to time (PERMANOVA: R^2^ = 0.344, *p* = 0.001; beta-dispersion: *p* > 0.05), which explained 34.4% of the variation observed. This pattern was also observed in the ordination visualization, where a clear division between days is observed from day zero to 28, followed by a less clear temporal pattern from day 35 to 70 (Fig. 2B), suggesting diversification in all systems across time. The bacterial communities also segregated based on the type of system (PERMANOVA: R^2^ = 0.036, *p* = 0.001; beta-dispersion: *p* > 0.05), although this only explained 3.6% of the variation, which was not visually evident in the ordination visualization (Fig. 2C), nor when selecting other dimensions (Fig. S5B). However, multilevel pairwise PERMANOVA between the *P. inhibens* (WT) and the TDA-deficient mutant systems revealed that the community composition was influenced by the ability to produce TDA (PERMANOVA: R^2^ = 0.024, *p* = 0.003). This points to the fact that the presence of *P. inhibens* (WT) was only a minor driver of shaping the community composition.

We speculated that the small effect of the *P. inhibens* (WT) could be explained by each biological replicate (lineage) having different evolutionary trajectories, potentially owing to stochasticity derived from small differences in starting conditions. Indeed, the PERMANOVA revealed that lineage could explain 15.6% of the variance observed (PERMANOVA: R^2^ = 0.156, *p* = 0.001; beta-dispersion: *p* > 0.05), and that the effect of lineage depended on time (PERMANOVA: R^2^ = 0.079, *p* = 0.001; beta-dispersion: *p* > 0.05). We, therefore, tracked the trajectories of the bacterial community composition of each lineage (Fig. S6), which revealed that especially lineage three in the *P. inhibens* (WT) system diversified from the other *P. inhibens* (WT) lineages from day 21 to 70. This could be explained by two ASVs, *Marinobacter* ASV25 (*r* = 0.59, *p* = 0.001) and *Sulfitobacter* ASV14 (*r* = 0.6, *p* = 0.001), having a relative higher abundance, and *Marivita* ASV5 (*r* = 0.58, *p* = 0.001) having a relative lower abundance at these days compared to the remaining *P. inhibens* (WT) system lineages (Table S3). This indicated that lineages within the three systems exhibited distinct temporal patterns in community composition.

### Relative abundance trajectories of single ASVs reveal that a subset of ASVs is influenced by the presence of *Phaeobacter*

Since we observed temporal variability between lineages within the same system (Fig. S6), elucidating differences in ASVs trajectories between systems can be difficult. Therefore, to clarify the underlying dynamics in the microbial communities, we used linear mixed effect modeling (random effect being the lineage) to identify significant relative abundance trajectories of ASVs between the three systems, only including ASVs that were present in more than one sample.

**Fig. 3:**
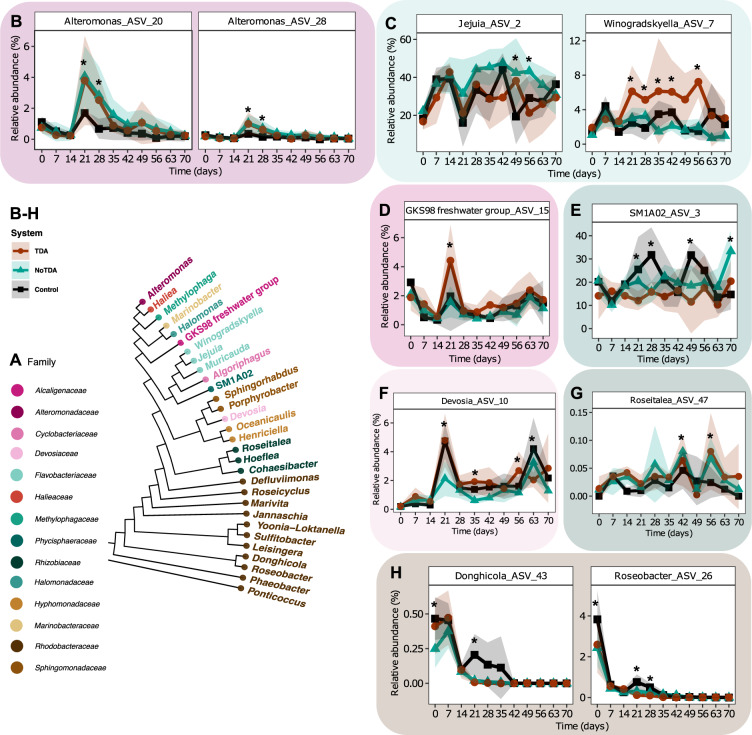
A subset of ASVs show temporal different abundance trajectories when exposed to *Phaeobacter inhibens*. **A** Phylogenetic tree with identified genus names of ASVs analyzed. Genera names are colored by family. **B**–**H** Relative abundance trajectories of significant different ASVs, identified using linear mixed effect models (LMM). Asterisks represent timepoints with significant differences between two or more systems (*p* < 0.05, LMM and EMM). Specific significant differences are listed in Table S4. Solid lines represent mean relative abundance and ribbons standard deviation of ASVs classified at genus level as **B** *Alteromonas*, **C** *Jejunia, Winogradskyella*, **D** GKS98 freshwater group, **E** SM1A02, **F** *Devosia*, **G** *Roseitelea*, and **H** *Donghicola* and *Roseobacter* (*N* = 4).

Out of these 40 ASVs (Fig. S7), ten ASVs displayed significant different trajectories between the three systems at least one timepoint (*p* < 0.05, EMMs, Fig. 3B–H, Table S4). After the first batch transfer at day 21 to 28, the two *Alteromonas* ASVs 20 and 28 gained a significant higher relative abundance in the systems with the *P. inhibens* (WT) (day 21: *p* = 0.005 and *p* = 0.001; day 28: *p* = 0.0004 and *p* = 0.001, EMMs) and the TDA-deficient mutant (day 21: *p* = 0.014 and *p* = 0.005; day 28: *p* = 0.002 and *p* = 0.009, EMMs) compared to the control system (Fig. 3B), indicating that the higher relative abundances of *Alteromonas* ASVs were primarily an effect of the addition of *P. inhibens*, rather than the ability of the bacterium to produce TDA. In contrast, an ASV classified as *Donghicola* (ASV43) disappeared faster from the two *P. inhibens* systems after day 21 (WT: *p* < 0.0001 and TDA-deficient mutant: *p* < 0.0001, compared to the control system, EMMs), whereas the ASV remains in the control system until day 35 before it goes extinct (Fig. 3H). Besides the before mentioned ASVs, five ASVs displayed significant differences at day 21, including *Winogradskyella* ASV7 (Fig. 3C), GKS98 freshwater group ASV15 (Fig. 3D), SM1A02 ASV3 (Fig. 3E), *Devosia* ASV10 (Fig. 3F), *Roseitalea* ASV47 (Fig. 3G) and *Roseobacter* ASV26 (Fig. 3H). However, the differences at day 21 is not similar between ASVs (Fig. 3). These changed dynamics following day 21 indicate that the batch transfer resets the communities differentially depending on whether *P. inhibens* has been added previously to the systems. Interestingly, the *Winogradskyella* ASV7 displayed higher relative abundance in communities with *P. inhibens* (WT) compared to the two other systems after the batch transfer at day 21 until day 56 (Fig. 3C), where some days the relative abundance was significant different (*p* < 0.05, EMMs). This indicates that the *Winogradskyella* strain is positively influenced by the presence of a TDA-producing *Phaeobacter*. Altogether, the addition of *Phaeobacter*, capable of producing TDA or not, can influence the relative abundance trajectories of strains present in the microalgae community over time.

### The absolute abundance profiles of *Phaeobacter* are different across systems

**Fig. 4:**
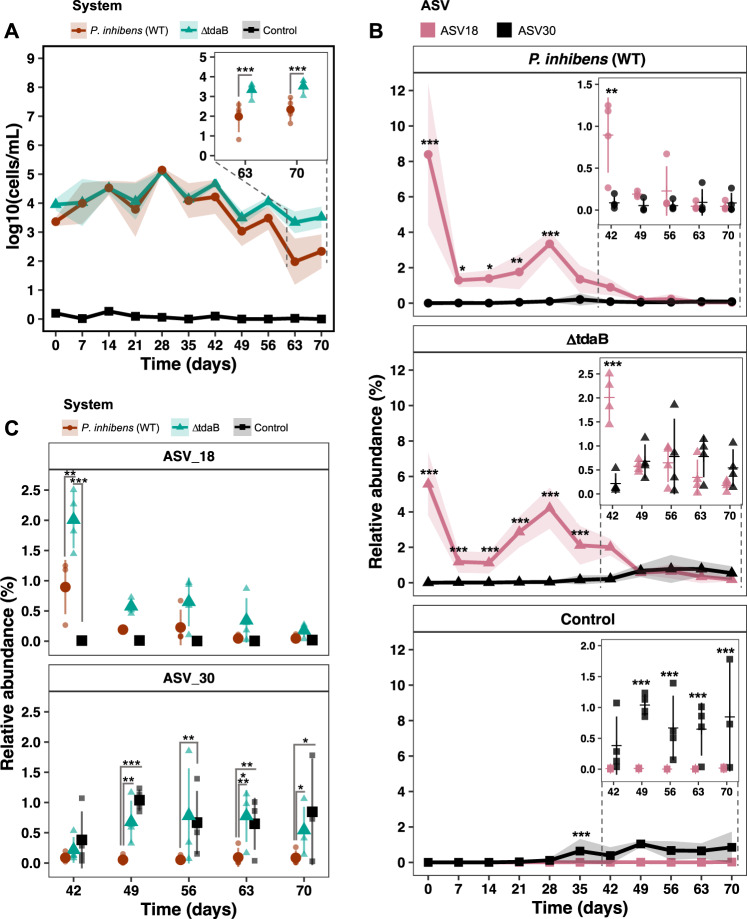
Absolute and relative abundance trajectories of *Phaeobacter* ASVs in the microbiome of the microalga *T. suecica*. **A** The absolute abundances of bacteria belonging to the *Phaeobacter* genus measured by qPCR using genus-specific primers. Symbols represent means and colored ribbons the standard deviation. Asteriks denotes significance level (*p* < 0.05, LMM and EMM). (*N* = 4). **B** Relative abundance of ASVs classified as *Phaeobacter* at genus level in the three systems. Points represent means and colored ribbons the standard deviation. In the time subset plots, horizontal lines represent means, filled symbols replicates and error bars the standard deviation. Asteriks denotes significance level (*p* < 0.05, LMM and EMM). (*N* = 4). **C** Comparisons of the two *Phaeobacter* ASVs: ASV18 and ASV30 between the three systems. Filled symbols represent means, transparent symbols replicates and error bars the standard deviation. Asteriks denotes significance level (*p* < 0.05, LMM and EMM). (*N* = 4).

We speculated whether the previous observations were caused by the elevated inoculum of *Phaeobacter* or by continued interaction over-time with *Phaeobacter* strains. We therefore sought to quantify the abundance of *Phaeobacter* by qPCR using genus specific primers [18]. There was a significant effect of time on the absolute abundance of *Phaeobacter* (*p* < 2.2e–16, LMM), since the level of *Phaeobacter* increased from log 3.4 ± 0.2 and 3.95 ± 0.2 CFU mL^−1^ in the *P. inhibens* (WT) and TDA-deficient mutant system, respectively, at day zero to approx. log 5 CFU mL^−1^ at day 28, followed by a decrease to log 2–3 CFU mL^−1^ (Fig. 4A). Thus, the level of *Phaeobacter* in the *P. inhibens* (WT) and TDA-deficient mutant system followed similar dynamics until day 56–70, where the level of *Phaeobacter* in the TDA-deficient mutant system was significantly higher than in the *P. inhibens* (WT) system (*p* < 0.05, EMMs). The qPCR approach also detected a low level of *Phaeobacter* (log 0.2 ± 0.26 CFU mL^−1^) being present in the control system at day zero, however, the abundance of this *Phaeobacter* population remained between log 0.2 ± 0.26 and 0 CFU mL^−1^, whereas levels of *Phaeobacter* in the *P. inhibens* (WT) and TDA-deficient mutant systems remained higher (*p* < 0.0001, EMMs), indicating that both *Phaeobacter* strains established themselves in the system.

### Expression of *tdaC* in the algal experimental setup

Differences in community composition were seen between the systems exposed to *P. inhibens* (WT) and the TDA-deficient mutant, however, it is not known if TDA is actually produced in the system. We have previously not been able to detect TDA chemically in algal systems [17] and attempts in this study were also not successful (data not shown). We therefore decided to use gene expression as a proxy for the actual production and developed a RT qPCR protocol for detecting the expression of the *tda*C gene. *tda*C transcripts were detected in three out of four replicates (684 ± 854 transcripts mL^−1^) in the algal cultures, to which the *P. inhibens* (WT) was added. *tdaC* transcripts were also detected in the TDA-deficient mutant algal cultures in two out of four replicates (1137 and 2336 transcripts mL^−1^). The *tda*C gene was detected in all *P. inhibens* (WT) and TDA-deficient mutant samples at day one, four, and seven, and ranged from log 4.9–5.5 genes mL^−1^, day one being slightly higher than day four and seven. The TDA-deficient mutant has an insert in the *tdaB* gene and does not, in pure cultures, produce TDA. To ensure that the detection of *tdaC* transcripts in the algal cultures, to which we added the TDA-deficient mutant was not due to contamination, we PCR-amplified the *tda*B gene from the samples and this was only detected in algal cultures, to which the *P. inhibens* (WT) was added.

### Indigenous *Phaeobacter* ASV gains footing when TDA-producing *Phaeobacter* are not present

Finding bacteria belonging to the *Phaeobacter* genus in the control system led us to query whether these indigenous *Phaeobacter* were also present in the manipulated systems, and if so, at what levels. Relating unique ASVs to specific bacterial strains, or even species, can be difficult, if not misleading [57]. However, most species within the *Phaeobacter* genus can be distinguished by the 16S rRNA gene, as most species have distinct 16S alleles, including *P. inhibens* [58]. Out of the 46 ASVs present in the communities, two ASVs were classified as *Phaeobacter* at genus level (Fig. S8). The two *Phaeobacter* ASVs were identified as ASV18 matching *P. inhibens*, representing both the wildtype and the TDA-deficient mutant, and ASV30 matching another *Phaeobacter*, which has two nucleotide substitutions relative to *Phaeobacter* ASV18 (Fig. S8).

The relative abundance of ASV18 was significantly different between the three systems, between days and the difference between systems depended on the day (*p* < 2.2e–16, LMM, Fig. S7). The relative abundance profile of ASV18 from day zero to 35 were higher in the manipulated systems compared to the control (*p* < 0.05, EMMs, Fig. 4B). At day 42, the relative abundance decreased in the *P. inhibens* (WT) system (Fig. 4C), resulting in no significant difference between the *P. inhibens* (WT) and control system (*p* = 0.06, EMMs), but there was a significantly higher relative abundance of ASV18 in the TDA-deficient mutant system compared to both the *P. inhibens* (WT) and control (*p* = 0.01 and *p* = 2.1e–6, respectively, EMMs). In the remaining timepoints (day 49 to 70), no significant differences were found between either of the systems (*p* > 0.05, EMMs, Fig. 4C).

There was also a significant effect of time (*p* < 2.2e–16, LMM) on ASV30, and a significant difference between the systems (*p* = 0.008, LMM), which clearly depended on time (*p* = 2.1e–06, LMM, Fig. S7). The relative abundance dynamics of ASV30 were different from that of ASV18 in all systems (Figs. S7 and 4B). From day zero to 42 the relative abundance of ASV30 were below 1.6% in the control system, 0.6% in the TDA-deficient mutant system, and below 0.8% in the *P. inhibens* (WT), and therefore no significant differences were found between systems (*p* > 0.05, EMMs). From day 49 to 70, the relative abundances of ASV30 were significantly lower in the *P. inhibens* (WT) system compared to control system (*p* < 0.05, EMMs, Fig. 4C) and the TDA-deficient mutant system (*p* < 0.05, EMMs, Fig. 4C), except from day 56, where the level of ASV18 was similar between the TDA-deficient mutant and *P. inhibens* (WT) system (*p* = 0.06, EMMs, Fig. 4C). In contrast, no significant differences were found between the TDA-deficient mutant and control system in the same period (*p* > 0.05, EMMs, Fig. 4C), indicating that the relative abundance of ASV30 was the same in the two systems.

The significant differences observed in absolute abundance of the *Phaeobacter* genus between the *P. inhibens* (WT) and TDA-deficient mutant system in the late phase (Fig. 4A) could be explained by a higher relative abundance of ASV30 in the TDA-deficient mutant system (Fig. 4C). In fact, ASV18 co-existed with ASV30 for a period in the TDA-deficient mutant system, a pattern not observed in the *P. inhibens* (WT) system (Fig. 4B, C). In comparison, ASV30 was barely observed in the *P. inhibens* (WT) system at any time point, hence the most dominant *Phaeobacter* population can be assigned to ASV18 (Fig. 4B, C). Altogether these observations indicated that the addition of *Phaeobacter* (both *P. inhibens* (WT) and TDA-deficient mutant) suppressed the naturally occurring *Phaeobacter*.

Since the TDA-producing *P. inhibens* depleted faster over time compared to the TDA-deficient mutant, one could speculate whether this strain experienced a stronger selection pressure. To determine if the selective pressure was based on mutational changes, we isolated 48 isolates of TDA-producing *P. inhibens* (WT) at day 35, 63, and 70 for whole genome sequencing. At these days, we estimated that *P. inhibens* reached approximately 38.5, 69.3, and 75 generations, respectively. However, we did not observe any consistent mutation or pattern that indicated relevant genetic adaptions to the microalgal community or host (Table S5).

## Discussion

Understanding the role and influence of microbial secondary metabolites in microbiomes is crucial to the study of microbial ecology and evolution of microbial communities. Previous studies have predominantly studied the effect of a secondary metabolite producer on composition of microbial communities on a short timescale (< eight days) [6, 18, 25, 26, 59], in high concentrations [6, 18, 25, 26] and typically not tested the specific effects of the secondary metabolite by including isogenic mutants devoid of secondary metabolite production [6, 18]. Here, we addressed these issues by propagating microbial communities associated with the microalgae *T. suecica*, with or without presence of TDA-producing *P. inhibens* or its TDA-deficient mutant over 70 days.

Both the TDA-producing *P. inhibens* and the TDA-deficient mutant invaded the *T. suecica* microbiome and followed similar dynamics for the first 42 days, indicating that the ability to produce TDA did not affect the competitiveness of *P. inhibens* when invading the microbial community. *P. inhibens* is an effective colonizer of biotic and abiotic surfaces and can successfully invade marine environments such as mixed-species biofilms on an abiotic surface [60], on the macroalgae *Ulva australis* [15] and on the marine diatom *Thalassiosira rotula* [61]. This invasiveness has previously, in part, been attributed to TDA and its antibacterial function [26], however, our results suggest that the colonization and establishment in a microbial community does not depend on the ability of *P. inhibens* to produce TDA. Similarly, in a short-term study, no difference was observed in colonization or initial attachment between *P. inhibens* and a biofilm dispersant variant strain of *P. inhibens* with reduced antimicrobial activity in the *T. rotula* microbiome [26] or on abiotic surfaces [62].

Numbers of both the TDA-producing *P. inhibens* and the TDA-deficient mutant declined over time in the *T. suecica* system, with the TDA-producing *P. inhibens* disappearing faster than its TDA-deficient counterpart. Thus, the presence of *Phaeobacter* in the algal microbiome is not facilitated by the ability to produce TDA. The disappearance of the TDA-producer could be caused by competition sensing and interference competition [1, 63], leading to competitive exclusion by other competing microorganisms in the *T. suecica* microbiome. Competing strains must overlap in their ecological niche, e.g., in resource use, to be competitors [64], using the same logic as in Darwin’s naturalization hypothesis, where invaders will be less successful in communities that contain organisms that occupy the same niche as the invaders, e.g., are often close relatives due to the shared evolutionary route [65]. Therefore, competing strains do not necessarily need to be distantly related species but can differ by only a single mutation [64].

Interestingly, we found that after day 42, another native *Phaeobacter* population gained a footing in the *T. suecica* microbiome, but only in the microalgae cultures, to which the TDA-producing *P. inhibens* was not added. This means that non-TDA-producing *P. inhibens* can coexist with other *Phaeobacter* species/strains, and TDA-producing *P. inhibens* may experience strong competition due to the presence of the other *Phaeobacter* population. Since neither of the two *Phaeobacter* ASVs were abundant after day 42 in the microalgae cultures treated with *P. inhibens* (WT), we speculate that the TDA-producer was able to outcompete the native *Phaeobacter* population within the first 42 days. In a recent microalgae mesocosm experiment, a taxonomically similar, but functionally distinct, *Phaeobacter* strain became established as the inoculated *Phaeobacter gallaeciensis* disappeared from the mesocosm after 17 days [66]. This supports our observations that inoculated *Phaeobacter* strains hardly co-exist with native *Phaeobacter* species/strains in microalgae microbiomes. Previous studies have demonstrated that close relatives often are the most affected by the antimicrobial secondary metabolite producer [6, 18, 25, 67], however, only a limited number of studies have addressed this on a species or even strain level [1, 26]. Our results reveal that intraspecies interactions might be crucial for understanding the effect of secondary metabolites on bacterial community dynamics.

The bacterial community compositions were only to a limited degree influenced by the presence of TDA-producing *P. inhibens*, with only a minor effect on a subset of species/strains, with different taxonomic origin. We did not observe clear inhibition of other strains as an effect of the presence of the TDA-producer, instead, we observed that a *Winogradskyella* ASV were present in significantly higher relative abundance in communities treated with the TDA-producing *P. inhibens* (WT). This effect was also observed in a previous microalgae microbiome, where a *Winogradskyella* OTU had higher relative abundance upon the addition of TDA-producing *P. inhibens* [66]. *Winogradskyella* is a widespread marine genus within the family *Flavobacteriaceae* – a major microalgae-associated bacterial family [68, 69]. What causes the higher relative abundance of *Winogradskyella* in the *P. inhibens* (WT) treated systems is not uncovered, since *Phaeobacter* and *Winogradskyella* interactions have not been investigated. However, several members of the *Flavobacteriaceae* family are tolerant to TDA [70, 71], thus the increase could be an indirect effect of the reduction of bacteria that compete for the same niche as *Winogradskyella*, but who are sensitive to TDA. Similarly, two ASVs classified as *Alteromonas* at genus level increased in relative abundance in systems with addition of *P. inhibens* in the period following the first propagation. Previous studies addressing the effect of pure TDA or TDA-producing *Phaeobacter* on marine microbiomes have also observed that bacteria within Alteromonadales or *Alteromonadaceae* (order and family of *Alteromonas*) are affected [25, 26]. Yet, the effect is not consistent since some studies have reported reductions [18, 25] and others increases [25] within these groups, due to the presence of TDA or TDA-producing bacteria. Besides, species belonging to the *Alteromonas* genus are known to be TDA-tolerant to a higher degree than other community members in a TDA-producer environment [71], and hypothesized to be a key species influencing the inter-OTU relationship in the microbiome of the microalgae *Nanochroloropsis salina* [25]. However, in our study the alteration of *Alteromonas* strains/species were similar in the systems treated with TDA-producing *P. inhibens* and the TDA-deficient mutant, thus the effect could not be assigned the ability to produce TDA.

Altogether, only subtle differences were observed between the communities, corroborating previous *in vitro* community studies also reporting that alterations in marine communities are subtle at higher taxonomic levels, and tend to be species-, if not strain-specific [6, 18, 25, 26]. Furthermore, Harrington and colleagues [71] observed a high degree of TDA tolerance in non-TDA-producing bacterial isolates from marine eukaryote-associated microbiomes, thus one could speculate that the *T. suecica* microbiome is already adapted to the presence of TDA-producing bacteria, if the native *Phaeobacter* ASV is capable of producing TDA. If *P. inhibens* uses TDA as a broad-range antibiotic, a decrease in community richness or diversity could be expected, however, we did not observe this. In fact, the addition of *P. inhibens* to an oyster system had the opposite effect [18], indicating that TDA could serve other functions or may simply not be produced. However, we did detect *tdaC* transcripts from the *P. inhibens* (WT) after 24 h, indicating that TDA could be produced in this system, which makes this an ecological relevant model system for investigating the effect of TDA-producing *Phaeobacter* on microbial community composition in *T. suecica* microbiome. We are aware that the *tdaC* gene might not be essential for the production of TDA by *P. inhibens* [72], however, *tdaC* is in the same operon as *tdaD-E* [73], which both are essential for TDA production [72]. Additionally, we show that *tdaB* genes were only present in *P. inhibens* (WT) system, indicating the presence of TDA-producing *Phaeobacter*, while there were none present in the TDA-deficient mutant and control system. Thus, the *tdaC* transcripts detected in the *P. inhibens* (WT) system can be used as an indication for TDA production. *tdaC* transcripts were only detected at day one which could be an indication of TDA production during attachment to surfaces and biofilm formation as seen for *Tritonibacter mobilis* strain F1926 [74], as growth of *P. inhibens* (WT) was not observed between day one and four as well as day seven. However, we cannot rule out that TDA could be produced throughout the cultivation period due to our limit of detection of *tdaC* transcripts in this system (>165 transcripts mL^−1^ detected from log 5.56 *P. inhibens* mL^−1^).

Previous microbiome studies of TDA or TDA-producing *Phaeobacter* have typically used short term exposure (< eight days) [18, 25]. Here, we demonstrate that TDA-producing *P. inhibens* also has an effect over longer time in a host-associated microbiome. More explicitly, we show that dynamics of closely related taxa differed between communities over time. These community dynamics would not have been observed on a short timescale, thus emphasizing the importance of considering the temporal scale and experimental setup when studying the response of complex microbial communities to biotic perturbations. Since the TDA-producing *P. inhibens* was depleted faster than its TDA-deficient counterpart, we hypothesized that this population was exposed to stronger competition and therefore higher selection pressure. Whole genome analysis of persisting TDA-producing *P. inhibens* isolates in the microbiome did, however, not reveal any selective sweeps or consistent genetic adaptions to the environment. It has been suggested that rapid adaption and evolution of an invader is a key factor that contributes to its invasion success [75]. One could speculate that the TDA-producing *P. inhibens* failed to adapt to the present bacterial community or that vital mutations will not appear until later.

In conclusion, TDA-producing *P. inhibens* can affect the microbial population dynamics in a host-associated microbiome. The invasion of *P. inhibens* is not dependent on its ability to produce TDA, and both a TDA-producing wild type and a TDA-deficient mutant persisted in the communities, following the same dynamics, for the first 42 days. Subsequently, both *P. inhibens* strains declined in abundance towards day 70, with the TDA-producing strain aiming towards extinction more rapidly. As the added *P. inhibens* strains declined in abundance, a closely related *Phaeobacter* ASV appeared, which only could co-exist with the non-TDA producing strain. Evidently, our study shows that strain-level studies, using a relevant natural occurring microbiome, are relevant to study the influence of secondary metabolites on community dynamics. Hence, these findings, supports the emerging view that strains are the most dynamic and interactive units of microbiomes [76, 77].

## Supplementary information

Suppl Figures

Suppl Tables

## Data Availability

The raw sequence reads obtained in this study have been deposited in the Sequencing Read Archive under BioProject number PRJNA795303. Remaining data and scripts can be obtained at https://github.com/nh91/NCLTEE.
