# Supplementary material for: The ability of Phaeobacter inhibens to produce tropodithietic acid influences the community dynamics of a microalgal microbiome
Source: ISME Commun. 2022 Nov 3;2:109. doi: 10.1038/s43705-022-00193-6 (PMC9723703; doi:10.1038/s43705-022-00193-6)
Supplement: Supplementary file 1 — Suppl Figures [file 43705_2022_193_MOESM1_ESM.docx]

**Supplementary figures**

**Figure S1. Core microbiome genera.** Relative abundance of top 20 bacterial genera in the *Tetraselmis suecica* microbiome during a 70-day co-cultivation experiment. *Phaeobacter* was excluded from the analysis. Bars represent mean relative abundance across all samples. Standard deviation is shown as error bars. *N* = 12.

**Figure S2.** *Tetraselmis suecica* abundances in a seven day long co-cultivation experiment with *P. inhibens* (WT), a TDA-deficient mutant (△tdaB), and alone (Control). Filled circles represent mean abundance, transparent points biological replicates and error bars standard deviation. *N* = 4.

**Figure S3. Heat Tree of genera composition of the 46 ASVs found in the *T. suecica* bacterial community at day 42.** Each colored tree represents pairwise comparisons for the three systems*, P. inhibens* (WT), TDA-deficient mutant (ΔtdaB), and Control. The grey tree on the lower left is a taxonomic reference for the smaller unlabeled trees. The color of each taxon reflects differential abundance between the two systems being compared with colors determined by the log2 ratio of median proportions of reads observed in each system. The size of the nodes relates to the number of ASVs found within the given taxonomic group. *N* = 4.

**Figure S4. A)** Stress values for ten dimensions in (k = 10) in nonmetric multidimensional scaling (nMDS). **B)** Shepard plot for nonmetric multidimensional scaling (nMDS) results. The red solid line represents the linear relationship between original observed Bray-Curtis dissimilarities between samples and nMDS ordination distances. Two correlation statistics on the goodness of fit is shown. Non-metric fit (R^2^) is based on the stress-value and the linear fit (R^2^) on squared correlation between fitted values and ordination distances.

**Figure S5.** Non-metric multidimensional scaling (NMDS) with three dimensions (1-3) on Bray-Curtis distances of ASV composition. Each point represent a sample colored by **A)** Time (day 0-70) and **B)** System (*P. inhibens* (WT), △tdaB, and Control). Stress = 0.09986.

|  | **Figure S6.** Non-metric multidimensional scaling (NMDS) on Bray-Curtis distances of ASV composition. The first two of three dimensions are shown. Symbols represent samples from each lineage (1-4) and coloured lines represent lineage trajectories (1-4). Each sample point is labelled with day. The grey lines indicate significant correlations of ASVs (*p =* 0.001, *r* > 0.49) with the ordination using *envfit* function from the R-package ‘*vegan’*. The ordination is divided into **A)** samples from the *P. inhibens* (WT) system, **B)** △tdaB system, and **C)** Control system. Stress = 0.1170633. |
| --- | --- |

**Figure S7.** Relative abundance trajectories of ASVs. Linear mixed effect model (random effect being lineage) were used to test the effect of time and system on the relative abundance profiles. P-values for the fixed variables systems (P*. inhibens* (WT), △tdaB, and Control), day (0-70) and the interaction term (System:day) is printed for each ASV. ASVs that are significant between systems is printed in bold. Solid lines represent mean relative abundance and ribbons standard deviation. *N* = 4

**Figure S8.** Alignment of reference sequences for ASV sequences ASV18 and ASV30 classified as *Phaeobacter* at genus level. Sequences were extracted from the *phyloseq* object and aligned in CLC Main Workbench 8 using default settings. Two nucleotide differences are observed at position 232 and 244.
