# Supplementary material for: The ability of Phaeobacter inhibens to produce tropodithietic acid influences the community dynamics of a microalgal microbiome
Source: ISME Commun. 2022 Nov 3;2:109. doi: 10.1038/s43705-022-00193-6 (PMC9723703; doi:10.1038/s43705-022-00193-6)
Supplement: Supplementary file 2 — Suppl Tables [file 43705_2022_193_MOESM2_ESM.docx]

| **Table S1**. Numbers of reads from 16S amplicon sequencing (V3V4) passing through each step in the DADA2 pipeline. Sample names identify timpoint (T0-10), system (P = *P. inhibens* (WT), D = △tdaB and C = Control), and lineage (1-4). | | | | | | |
| --- | --- | --- | --- | --- | --- | --- |
| **Sample** | **Input** | **Filtered** | **DenoisedF** | **DenoisedR** | **Merged** | **Non-chimeric** |
| **T0-P1** | 463621 | 370440 | 369670 | 369376 | 364535 | 362942 |
| **T0-P2** | 267404 | 218979 | 218832 | 218654 | 217154 | 215140 |
| **T0-P3** | 199357 | 155731 | 155611 | 155634 | 154777 | 154397 |
| **T0-P4** | 241158 | 180008 | 179844 | 179853 | 178089 | 177306 |
| **T0-D1** | 1809 | 1330 | 1287 | 1311 | 1273 | 1271 |
| **T0-D2** | 423733 | 349106 | 348636 | 348620 | 344089 | 338492 |
| **T0-D3** | 2216 | 1786 | 1760 | 1768 | 1740 | 1735 |
| **T0-D4** | 694127 | 540893 | 540053 | 539186 | 531137 | 506894 |
| **T0-C1** | 598886 | 435620 | 435264 | 434938 | 429621 | 425000 |
| **T0-C2** | 333 | 229 | 204 | 219 | 194 | 194 |
| **T0-C3** | 87979 | 62137 | 61960 | 62018 | 61003 | 59048 |
| **T0-C4** | 154489 | 98876 | 98695 | 98736 | 98020 | 97083 |
| **Negative** | 78 | 52 | 22 | 26 | 10 | 10 |
| **T1-P1** | 408579 | 299256 | 298647 | 298795 | 295565 | 275413 |
| **T1-P2** | 192292 | 127909 | 127676 | 127683 | 126487 | 124079 |
| **T1-P3** | 208608 | 133409 | 132937 | 133227 | 131153 | 119704 |
| **T1-P4** | 121750 | 81983 | 81764 | 81842 | 80909 | 78159 |
| **T1-D1** | 87024 | 59878 | 59714 | 59752 | 59063 | 57161 |
| **T1-D2** | 105764 | 73285 | 73119 | 73138 | 72442 | 71548 |
| **T1-D3** | 215500 | 87389 | 87109 | 87171 | 85110 | 76036 |
| **T1-D4** | 177505 | 150593 | 150179 | 150376 | 148858 | 138271 |
| **T1-C1** | 320942 | 277062 | 276546 | 276572 | 274267 | 263294 |
| **T1-C2** | 88067 | 73132 | 72952 | 72947 | 72512 | 71589 |
| **T1-C3** | 175370 | 148580 | 148068 | 148293 | 146423 | 135230 |
| **T1-C4** | 177479 | 142190 | 141753 | 141954 | 140733 | 134538 |
| **Negative** | 664 | 560 | 494 | 512 | 459 | 459 |
| **T2-P1** | 316117 | 263616 | 262972 | 263242 | 261330 | 248338 |
| **T2-P2** | 131228 | 106431 | 106289 | 106193 | 105492 | 102656 |
| **T2-P3** | 266340 | 226911 | 226506 | 226570 | 224569 | 218092 |
| **T2-P4** | 118627 | 98911 | 98862 | 98870 | 98676 | 98405 |
| **T2-D1** | 277559 | 219821 | 219513 | 219571 | 217748 | 199461 |
| **T2-D2** | 402980 | 328309 | 327581 | 327843 | 325053 | 297455 |
| **T2-D3** | 243166 | 186391 | 186130 | 185952 | 184583 | 180242 |
| **T2-D4** | 499216 | 349918 | 349154 | 349349 | 345475 | 297523 |
| **T2-C1** | 4444 | 3158 | 3133 | 3151 | 3086 | 3075 |
| **T2-C2** | 243165 | 198766 | 198380 | 198575 | 197186 | 187286 |
| **T2-C3** | 382145 | 302831 | 301946 | 302151 | 297979 | 252583 |
| **T2-C4** | 235164 | 174557 | 173958 | 174175 | 170967 | 145418 |
| **T3-P1** | 618551 | 484463 | 483689 | 483740 | 480110 | 459103 |
| **T3-P2** | 174570 | 136581 | 136016 | 136263 | 133104 | 111419 |
| **T3-P3** | 194448 | 145979 | 145698 | 145715 | 144220 | 134730 |
| **T3-P4** | 108992 | 78845 | 78600 | 78683 | 77495 | 75513 |
| **T3-D1** | 199692 | 148338 | 147982 | 147962 | 145865 | 137021 |
| **T3-D2** | 250708 | 190425 | 190123 | 190214 | 188740 | 184590 |
| **T3-D3** | 167190 | 121334 | 121119 | 121081 | 119826 | 118205 |
| **T3-D4** | 204243 | 142192 | 142008 | 142060 | 141219 | 138558 |
| **T3-C1** | 238544 | 173533 | 173183 | 173252 | 171165 | 166782 |
| **T3-C2** | 467345 | 349549 | 348501 | 348918 | 345144 | 329120 |
| **T3-C3** | 21364 | 15687 | 15636 | 15641 | 15434 | 15291 |
| **T3-C4** | 233912 | 134325 | 133799 | 134018 | 130880 | 120301 |
| **Negative** | 535 | 465 | 360 | 380 | 334 | 334 |
| **T4-P1** | 429243 | 373915 | 373030 | 373292 | 370029 | 345263 |
| **T4-P2** | 200916 | 164001 | 163699 | 163659 | 162345 | 157562 |
| **T4-P3** | 77199 | 65297 | 65040 | 65143 | 64273 | 60883 |
| **T4-P4** | 197856 | 154511 | 154058 | 154234 | 152511 | 143944 |
| **T4-D1** | 391638 | 317806 | 317451 | 317306 | 314420 | 308706 |
| **T4-D2** | 191645 | 157720 | 157192 | 157343 | 155671 | 144338 |
| **T4-D3** | 192440 | 154818 | 154468 | 154505 | 153354 | 149855 |
| **T4-D4** | 207094 | 175327 | 175219 | 175229 | 174249 | 174109 |
| **T4-C1** | 405904 | 337846 | 337170 | 337273 | 333732 | 299820 |
| **T4-C2** | 249179 | 195167 | 194760 | 194889 | 192662 | 182552 |
| **T4-C3** | 219682 | 179321 | 179008 | 178902 | 177353 | 172196 |
| **T4-C4** | 358883 | 275200 | 274889 | 274647 | 272703 | 265999 |
| **T5-P1** | 399399 | 273344 | 272761 | 272756 | 269869 | 262576 |
| **T5-P2** | 155435 | 109702 | 109546 | 109530 | 108949 | 107027 |
| **T5-P3** | 185344 | 151139 | 150909 | 150916 | 149671 | 145615 |
| **T5-P4** | 99072 | 76912 | 76824 | 76839 | 76503 | 76142 |
| **T5-D1** | 401623 | 295480 | 295170 | 294697 | 292522 | 287504 |
| **T5-D2** | 442953 | 320068 | 319543 | 319749 | 317710 | 311596 |
| **T5-D3** | 209668 | 150843 | 150637 | 150659 | 149426 | 147081 |
| **T5-D4** | 531191 | 370734 | 370491 | 370136 | 366535 | 357111 |
| **T5-C1** | 164608 | 102190 | 101992 | 102029 | 101051 | 99338 |
| **T5-C2** | 191147 | 130744 | 130511 | 130539 | 129407 | 124483 |
| **T5-C3** | 189264 | 133959 | 133675 | 133794 | 132601 | 127827 |
| **T5-C4** | 167099 | 108418 | 108262 | 108304 | 107677 | 106197 |
| **Negative** | 986 | 626 | 600 | 606 | 569 | 564 |
| **T6-P1** | 274534 | 183578 | 183283 | 183272 | 181723 | 173330 |
| **T6-P2** | 40341 | 26703 | 26594 | 26670 | 26390 | 26107 |
| **T6-P3** | 54652 | 37027 | 36865 | 36913 | 36202 | 35547 |
| **T6-P4** | 80183 | 32078 | 31922 | 32018 | 31296 | 30200 |
| **T6-D1** | 356177 | 293135 | 292801 | 292749 | 291075 | 284988 |
| **T6-D2** | 264781 | 224463 | 224076 | 224008 | 222494 | 215915 |
| **T6-D3** | 285571 | 230688 | 230409 | 230431 | 229203 | 225514 |
| **T6-D4** | 318557 | 261505 | 261195 | 261263 | 259848 | 254227 |
| **T6-C1** | 247290 | 192456 | 191938 | 192061 | 189388 | 173871 |
| **T6-C2** | 300236 | 242098 | 241824 | 241854 | 240844 | 237368 |
| **T6-C3** | 509181 | 407116 | 406627 | 406685 | 404003 | 379930 |
| **T6-C4** | 325977 | 256734 | 256220 | 256178 | 252614 | 218393 |
| **Negative** | 1857 | 1561 | 1473 | 1483 | 1425 | 1401 |
| **T7-P1** | 548964 | 445043 | 444753 | 444568 | 442302 | 426423 |
| **T7-P2** | 219759 | 173917 | 173733 | 173770 | 172887 | 170231 |
| **T7-P3** | 556 | 437 | 423 | 424 | 407 | 402 |
| **T7-P4** | 237547 | 187359 | 187215 | 187268 | 186680 | 185549 |
| **T7-D1** | 172856 | 125945 | 125836 | 125840 | 125263 | 124443 |
| **T7-D2** | 375213 | 279334 | 279191 | 279156 | 278015 | 275354 |
| **T7-D3** | 413061 | 335533 | 335211 | 335209 | 333623 | 325778 |
| **T7-D4** | 373709 | 293706 | 293510 | 293522 | 292310 | 290648 |
| **T7-C1** | 508418 | 396727 | 395974 | 396237 | 392116 | 359745 |
| **T7-C2** | 218932 | 120018 | 119839 | 119873 | 119159 | 118329 |
| **T7-C3** | 726314 | 544858 | 544458 | 543991 | 540846 | 525402 |
| **T7-C4** | 265691 | 194538 | 194474 | 194506 | 194112 | 193994 |
| **T8-P1** | 389852 | 286982 | 286438 | 286545 | 283846 | 261248 |
| **T8-P2** | 380432 | 280670 | 280319 | 280411 | 278782 | 269563 |
| **T8-P3** | 310128 | 243803 | 243406 | 243425 | 240415 | 226486 |
| **T8-P4** | 285922 | 208646 | 208327 | 208354 | 206629 | 201471 |
| **T8-D1** | 283719 | 196466 | 196137 | 196264 | 194729 | 188867 |
| **T8-D2** | 336042 | 244507 | 244119 | 244141 | 241601 | 228445 |
| **T8-D3** | 67751 | 50505 | 50411 | 50419 | 49782 | 48731 |
| **T8-D4** | 258572 | 203605 | 203253 | 203232 | 201258 | 188722 |
| **T8-C1** | 262311 | 161277 | 160942 | 161030 | 159462 | 156322 |
| **T8-C2** | 152576 | 123833 | 123473 | 123615 | 122048 | 114503 |
| **T8-C3** | 190629 | 159605 | 159093 | 159225 | 157227 | 141992 |
| **T8-C4** | 250249 | 198614 | 198252 | 198257 | 196641 | 188019 |
| **Negative** | 330 | 275 | 189 | 183 | 158 | 154 |
| **T9-P1** | 285218 | 216641 | 216309 | 216242 | 214506 | 201654 |
| **T9-P2** | 368087 | 296140 | 295771 | 295801 | 293915 | 282176 |
| **T9-P3** | 200959 | 155859 | 155641 | 155664 | 155011 | 153880 |
| **T9-P4** | 385830 | 296089 | 295856 | 295920 | 294624 | 292696 |
| **T9-D1** | 299436 | 245516 | 245103 | 245143 | 244099 | 243175 |
| **T9-D2** | 349532 | 272614 | 272341 | 272438 | 270950 | 266247 |
| **T9-D3** | 221857 | 172955 | 172606 | 172719 | 170886 | 163002 |
| **T9-D4** | 229245 | 180594 | 180290 | 180425 | 179480 | 175618 |
| **T9-C1** | 252442 | 193467 | 193107 | 193232 | 191776 | 188157 |
| **T9-C2** | 304309 | 217253 | 216872 | 216811 | 214240 | 195963 |
| **T9-C3** | 319191 | 236282 | 235900 | 235817 | 233800 | 223592 |
| **T9-C4** | 212716 | 167749 | 167561 | 167609 | 166852 | 164374 |
| **Negative** | 312 | 251 | 216 | 221 | 201 | 197 |
| **T10-P1** | 324849 | 240248 | 239816 | 240026 | 238259 | 225872 |
| **T10-P2** | 251145 | 142144 | 141748 | 141909 | 139905 | 132152 |
| **T10-P3** | 225070 | 166057 | 165703 | 165799 | 163683 | 157932 |
| **T10-P4** | 183175 | 128565 | 128313 | 128359 | 127090 | 122017 |
| **T10-D1** | 241304 | 161939 | 161687 | 161748 | 160672 | 158661 |
| **T10-D2** | 352088 | 239160 | 238675 | 238755 | 235618 | 216942 |
| **T10-D3** | 302822 | 231389 | 230897 | 230929 | 227531 | 209800 |
| **T10-D4** | 304468 | 214553 | 214271 | 214271 | 212611 | 203401 |
| **T10-C1** | 128975 | 78767 | 78558 | 78628 | 77650 | 75420 |
| **T10-C2** | 246 | 174 | 144 | 162 | 138 | 138 |
| **T10-C3** | 83601 | 57626 | 57270 | 57308 | 55413 | 48258 |
| **T10-C4** | 209141 | 153970 | 153735 | 153770 | 152706 | 148234 |
| **Negative** | 616 | 348 | 290 | 302 | 251 | 251 |
| **Total** | **33999951** | **25738532** | **25693722** | **25698204** | **25465765** | **24382203** |
| **Mean** | **242856.79** | **183846.66** | **183526.59** | **183558.60** | **181898.32** | **174158.59** |
| **SD(+/-)** | **148971.03** | **116603.13** | **116460.78** | **116421.90** | **115435.26** | **110439.31** |

| **Table S2.** Alpha diversity measures given as observed richness (ASV) for bacterial communities associated with *Tetraselmis suecica* treated with *Phaeobacter inhibens* (WT), *P. inhibens* TDA-deficient mutant (△tdaB) and f/2 (Control). The alpha diversity is based on 2-4 biological replicates over a period of 70 days (Time). | | | | | | |
| --- | --- | --- | --- | --- | --- | --- |
|  |  |  |  |  |  |  |
|  | *P. inhibens (WT)* | | *△tdaB* | | *Control* | |
| Time (day) | Replicates | Mean±SD | Replicates | Mean±SD | Replicates | Mean±SD |
| 0 | 4 | 34.8±2.9 | 2 | 37.5±0.7 | 3 | 33±3.6 |
| 7 | 4 | 34.5±2.4 | 4 | 32.3±3.2 | 4 | 32.8±2.1 |
| 14 | 4 | 34.3±2.2 | 4 | 36.5±0.6 | 3 | 33.7±1.2 |
| 21 | 4 | 36±2.2 | 4 | 35.8±1 | 4 | 34.3±4.3 |
| 28 | 4 | 33.5±4.5 | 4 | 34.5±2.4 | 4 | 36.3±1.7 |
| 35 | 4 | 28.8±5.1 | 4 | 30.8±0.5 | 4 | 29.8±3.3 |
| 42 | 4 | 26.8±2.4 | 4 | 32.3±0.5 | 4 | 32.3±4.4 |
| 49 | 3 | 28.3±5.1 | 4 | 28.3±2.6 | 4 | 27.3±4.7 |
| 56 | 4 | 28.8±3.1 | 4 | 27.8±1.3 | 4 | 27.5±3.1 |
| 63 | 4 | 27.3±1.5 | 4 | 27.5±2.5 | 4 | 28.3±3.8 |
| 70 | 4 | 27.8±2.8 | 4 | 25.8±2.6 | 3 | 23.3±3.2 |

| **Table S3**. Correlation coefficient (*r*) and p-values for ASV variables correlated with non-metric dimensional scaling ordination (nMDS). Only taxa with *r* > 0.5 and *p*-value < 0.05 are included. The values were generated using the function *envfit* from the R package 'vegan' (permutations = 1000). | | | | | | | |
| --- | --- | --- | --- | --- | --- | --- | --- |
|  |  |  |  |  |  |  |  |
| **Class** | **Order** | **Family** | **Genus** | **ASV** | ***r*** | **p-value** |  |
| Alphaproteobacteria | Rhodobacterales | *Rhodobacteraceae* | *Leisingera* | ASV_29 | 0.504 | 0.001 |  |
| Alphaproteobacteria | Rhodobacterales | *Rhodobacteraceae* | *Sulfitobacter* | ASV_14 | 0.598 | 0.001 |  |
| Alphaproteobacteria | Rhodobacterales | *Rhodobacteraceae* | *Yoonia-Loktanella* | ASV_44 | 0.602 | 0.001 |  |
| Alphaproteobacteria | Rhodobacterales | *Rhodobacteraceae* | *Jannaschia* | ASV_8 | 0.683 | 0.001 |  |
| Alphaproteobacteria | Rhodobacterales | *Rhodobacteraceae* | *Marivita* | ASV_5 | 0.576 | 0.001 |  |
| Gammaproteobacteria | Pseudomonadales | *Halieaceae* | *Haliea* | ASV_42 | 0.512 | 0.001 |  |
| Gammaproteobacteria | Pseudomonadales | *Marinobacteraceae* | *Marinobacter* | ASV_25 | 0.591 | 0.001 |  |
| Gammaproteobacteria | Pseudomonadales | *Marinobacteraceae* | *Marinobacter* | ASV_11 | 0.564 | 0.001 |  |
| Gammaproteobacteria | Pseudomonadales | *Marinobacteraceae* | *Marinobacter* | ASV_21 | 0.560 | 0.001 |  |
| Alphaproteobacteria | Rhizobiales | *Rhizobiaceae* | *Hoeflea* | ASV_6 | 0.511 | 0.001 |  |
| Alphaproteobacteria | Rhodobacterales | *Rhodobacteraceae* | *Donghicola* | ASV_43 | 0.588 | 0.001 |  |
| Alphaproteobacteria | Rhodobacterales | *Rhodobacteraceae* | *Ponticoccus* | ASV_12 | 0.610 | 0.001 |  |

| **Table S4.** Outcome from linear mixed effect (LMM) modelling and post hoc analysis with emmeans (EMM) for ASVs showing significant differences between the three systems *P. inhibens* (WT), TDA-deficient mutant (d-tdaB) and Control over time (day). P-values are adjusted with Bonferroni. Only days with *p* < 0.05 are shown. | | | | | | | |
| --- | --- | --- | --- | --- | --- | --- | --- |
|  |  |  |  |  |  |  |  |
| **System_pairwise** | **day** | **estimate** | **SE** | **df** | **t.ratio** | **p.value** | **ASV** |
| Control - d-tdaB | 42 | -2.9 | 7.2 | 46.1 | -0.4 | 1.000 | ***Jejuia* ASV2** |
| Control - WT | 42 | 15.3 | 7.2 | 46.1 | 2.1 | 0.120 | ***Jejuia* ASV2** |
| d-tdaB - WT | 42 | 18.2 | 7.2 | 46.1 | 2.5 | 0.046 | ***Jejuia* ASV2** |
| Control - d-tdaB | 49 | -31.9 | 7.7 | 52.8 | -4.2 | < 0.001 | ***Jejuia* ASV2** |
| Control - WT | 49 | -15.8 | 7.6 | 52.8 | -2.1 | 0.131 | ***Jejuia* ASV2** |
| d-tdaB - WT | 49 | 16.1 | 8.1 | 58.7 | 2.0 | 0.152 | ***Jejuia* ASV2** |
| Control - d-tdaB | 56 | -14.1 | 7.2 | 46.1 | -1.9 | 0.172 | ***Jejuia* ASV2** |
| Control - WT | 56 | 7.8 | 7.2 | 46.1 | 1.1 | 0.854 | ***Jejuia* ASV2** |
| d-tdaB - WT | 56 | 21.9 | 7.2 | 46.1 | 3.0 | 0.012 | ***Jejuia* ASV2** |
| Control - d-tdaB | 21 | 5.1 | 5.3 | 78.7 | 1.0 | 1.000 | **SM1A02 ASV3** |
| Control - WT | 21 | 13.4 | 5.3 | 78.7 | 2.5 | 0.042 | **SM1A02 ASV3** |
| d-tdaB - WT | 21 | 8.4 | 5.3 | 78.7 | 1.6 | 0.364 | **SM1A02 ASV3** |
| Control - d-tdaB | 28 | 15.8 | 5.3 | 78.7 | 3.0 | 0.012 | **SM1A02 ASV3** |
| Control - WT | 28 | 16.0 | 5.3 | 78.7 | 3.0 | 0.011 | **SM1A02 ASV3** |
| d-tdaB - WT | 28 | 0.2 | 5.3 | 78.7 | 0.0 | 1.000 | **SM1A02 ASV3** |
| Control - d-tdaB | 49 | 13.2 | 5.3 | 78.7 | 2.5 | 0.048 | **SM1A02 ASV3** |
| Control - WT | 49 | 19.7 | 5.7 | 82.9 | 3.4 | 0.003 | **SM1A02 ASV3** |
| d-tdaB - WT | 49 | 6.6 | 5.7 | 82.9 | 1.1 | 0.765 | **SM1A02 ASV3** |
| Control - d-tdaB | 70 | -19.7 | 5.7 | 82.9 | -3.4 | 0.003 | **SM1A02 ASV3** |
| Control - WT | 70 | -6.8 | 5.7 | 82.9 | -1.2 | 0.722 | **SM1A02 ASV3** |
| d-tdaB - WT | 70 | 12.9 | 5.3 | 78.7 | 2.4 | 0.053 | **SM1A02 ASV3** |
| Control - d-tdaB | 21 | -0.5 | 0.8 | 80.5 | -0.7 | 1.000 | ***Winogradskyella* ASV7** |
| Control - WT | 21 | -2.3 | 0.9 | 82.8 | -2.6 | 0.03 | ***Winogradskyella* ASV7** |
| d-tdaB - WT | 21 | -1.8 | 0.9 | 82.8 | -2.0 | 0.136 | ***Winogradskyella* ASV7** |
| Control - d-tdaB | 28 | -1.4 | 0.8 | 80.5 | -1.7 | 0.264 | ***Winogradskyella* ASV7** |
| Control - WT | 28 | -3.3 | 0.8 | 80.5 | -4.0 | < 0.001 | ***Winogradskyella* ASV7** |
| d-tdaB - WT | 28 | -1.9 | 0.8 | 80.5 | -2.3 | 0.075 | ***Winogradskyella* ASV7** |
| Control - d-tdaB | 35 | 2.0 | 0.8 | 80.5 | 2.5 | 0.049 | ***Winogradskyella* ASV7** |
| Control - WT | 35 | 0.2 | 0.9 | 82.7 | 0.3 | 1.000 | ***Winogradskyella* ASV7** |
| d-tdaB - WT | 35 | -1.8 | 0.9 | 82.7 | -2.0 | 0.139 | ***Winogradskyella* ASV7** |
| Control - d-tdaB | 42 | 1.6 | 0.8 | 80.5 | 1.9 | 0.174 | ***Winogradskyella* ASV7** |
| Control - WT | 42 | -0.7 | 0.9 | 82.7 | -0.8 | 1.000 | ***Winogradskyella* ASV7** |
| d-tdaB - WT | 42 | -2.3 | 0.9 | 82.7 | -2.6 | 0.035 | ***Winogradskyella* ASV7** |
| Control - d-tdaB | 56 | -0.4 | 0.8 | 80.5 | -0.5 | 1.000 | ***Winogradskyella* ASV7** |
| Control - WT | 56 | -5.7 | 0.8 | 80.5 | -7.0 | < 0.001 | ***Winogradskyella* ASV7** |
| d-tdaB - WT | 56 | -5.3 | 0.8 | 80.5 | -6.5 | < 0.001 | ***Winogradskyella* ASV7** |
| Control - d-tdaB | 70 | 1.4 | 0.9 | 82.9 | 1.6 | 0.337 | ***Winogradskyella* ASV7** |
| Control - WT | 70 | -0.7 | 0.9 | 82.9 | -0.8 | 1.000 | ***Winogradskyella* ASV7** |
| d-tdaB - WT | 70 | -2.1 | 0.8 | 80.5 | -2.5 | 0.039 | ***Winogradskyella* ASV7** |
| Control - d-WTB | 21 | 2.6 | 0.5 | 85.0 | 5.5 | <0.001 | ***Deviosa* ASV10** |
| Control - WT | 21 | -0.9 | 0.5 | 86.9 | -1.8 | 0.245 | ***Deviosa* ASV10** |
| d-WTB - WT | 21 | -3.5 | 0.5 | 86.9 | -6.9 | < 0.001 | ***Deviosa* ASV10** |
| Control - d-WTB | 35 | 0.8 | 0.5 | 85.0 | 1.6 | 0.331 | ***Deviosa* ASV10** |
| Control - WT | 35 | -0.5 | 0.5 | 85.0 | -1.2 | 0.756 | ***Deviosa* ASV10** |
| d-WTB - WT | 35 | -1.3 | 0.5 | 85.0 | -2.8 | 0.021 | ***Deviosa* ASV10** |
| Control - d-WTB | 56 | 0.5 | 0.5 | 85.0 | 1.0 | 1.000 | ***Deviosa* ASV10** |
| Control - WT | 56 | -1.1 | 0.5 | 85.0 | -2.3 | 0.071 | ***Deviosa* ASV10** |
| d-WTB - WT | 56 | -1.5 | 0.5 | 85.0 | -3.3 | 0.005 | ***Deviosa* ASV10** |
| Control - d-WTB | 63 | 2.0 | 0.5 | 86.9 | 3.9 | 0.001 | ***Deviosa* ASV10** |
| Control - WT | 63 | 3.2 | 0.5 | 86.9 | 6.3 | <0.001 | ***Deviosa* ASV10** |
| d-WTB - WT | 63 | 1.2 | 0.5 | 85.0 | 2.6 | 0.032 | ***Deviosa* ASV10** |
| Control - d-tdaB | 21 | -0.8 | 0.6 | 87.0 | -1.4 | 0.453 | **GKS98 freshwater group ASV15** |
| Control - WT | 21 | -4.7 | 0.6 | 88.5 | -8.0 | <0.001 | **GKS98 freshwater group ASV15** |
| d-tdaB - WT | 21 | -3.9 | 0.6 | 87.1 | -7.1 | <0.001 | **GKS98 freshwater group ASV15** |
| Control - d-tdaB | 21 | -1.5 | 0.5 | 84.1 | -2.9 | 0.014 | ***Alteromonas* ASV20** |
| Control - WT | 21 | -1.8 | 0.6 | 86.6 | -3.3 | 0.005 | ***Alteromonas* ASV20** |
| d-tdaB - WT | 21 | -0.4 | 0.6 | 87.1 | -0.7 | 1.000 | ***Alteromonas* ASV20** |
| Control - d-tdaB | 28 | -2.0 | 0.6 | 86.5 | -3.5 | 0.002 | ***Alteromonas* ASV20** |
| Control - WT | 28 | -1.9 | 0.5 | 81.9 | -4.0 | <0.001 | ***Alteromonas* ASV20** |
| d-tdaB - WT | 28 | 0.1 | 0.6 | 86.5 | 0.2 | 1.000 | ***Alteromonas* ASV20** |
| Control - d-tdaB | 0 | NA | NA | NA | NA | NA | ***Roseobacter* ASV26** |
| Control - WT | 0 | 1.5 | 0.2 | 87.0 | 9.9 | <0.001 | ***Roseobacter* ASV26** |
| d-tdaB - WT | 0 | NA | NA | NA | NA | NA | ***Roseobacter* ASV26** |
| Control - d-tdaB | 7 | 0.2 | 0.1 | 64.5 | 2.4 | 0.054 | ***Roseobacter* ASV26** |
| Control - WT | 7 | 0.1 | 0.1 | 64.5 | 0.9 | 1.000 | ***Roseobacter* ASV26** |
| d-tdaB - WT | 7 | -0.1 | 0.1 | 64.5 | -1.5 | 0.392 | ***Roseobacter* ASV26** |
| Control - d-tdaB | 21 | 0.4 | 0.1 | 64.5 | 5.6 | <0.001 | ***Roseobacter* ASV26** |
| Control - WT | 21 | 0.6 | 0.1 | 64.5 | 8.0 | <0.001 | ***Roseobacter* ASV26** |
| d-tdaB - WT | 21 | 0.2 | 0.1 | 64.5 | 2.3 | 0.067 | ***Roseobacter* ASV26** |
| Control - d-tdaB | 28 | 0.3 | 0.1 | 64.5 | 3.3 | 0.004 | ***Roseobacter* ASV26** |
| Control - WT | 28 | 0.4 | 0.1 | 64.5 | 5.0 | <0.001 | ***Roseobacter* ASV26** |
| d-tdaB - WT | 28 | 0.1 | 0.1 | 64.5 | 1.7 | 0.309 | ***Roseobacter* ASV26** |
| Control - d-tdaB | 21 | -0.4 | 0.1 | 81.9 | -3.2 | 0.005 | ***Alteromonas* ASV28** |
| Control - WT | 21 | -0.6 | 0.2 | 85.5 | -3.9 | 0.001 | ***Alteromonas* ASV28** |
| d-tdaB - WT | 21 | -0.2 | 0.2 | 86.4 | -1.0 | 0.987 | ***Alteromonas* ASV28** |
| Control - d-tdaB | 28 | -0.5 | 0.2 | 85.5 | -3.0 | 0.009 | ***Alteromonas* ASV28** |
| Control - WT | 28 | -0.5 | 0.1 | 78.9 | -3.7 | 0.001 | ***Alteromonas* ASV28** |
| d-tdaB - WT | 28 | 0.0 | 0.2 | 85.5 | 0.0 | 1.000 | ***Alteromonas* ASV28** |
| Control - d-tdaB | 0 | 0.2 | 0.0 | 87.0 | 4.4 | <0.001 | ***Donghicola* ASV43** |
| Control - WT | 0 | 0.0 | 0.0 | 87.0 | 0.1 | 1.000 | ***Donghicola* ASV43** |
| d-tdaB - WT | 0 | -0.2 | 0.1 | 87.0 | -3.9 | 0.001 | ***Donghicola* ASV43** |
| Control - d-tdaB | 21 | 0.2 | 0.0 | 87.0 | 4.9 | <0.001 | ***Donghicola* ASV43** |
| Control - WT | 21 | 0.2 | 0.0 | 87.0 | 5.2 | <0.001 | ***Donghicola* ASV43** |
| d-tdaB - WT | 21 | 0.0 | 0.0 | 87.0 | 0.3 | 1.000 | ***Donghicola* ASV43** |
| Control - d-tdaB | 14 | 0.0 | 0.0 | 85.2 | -1.8 | 0.208 | ***Roseitalea* ASV47** |
| Control - WT | 14 | 0.0 | 0.0 | 85.2 | -2.4 | 0.059 | ***Roseitalea* ASV47** |
| d-tdaB - WT | 14 | 0.0 | 0.0 | 84.0 | -0.6 | 1.000 | ***Roseitalea* ASV47** |
| Control - d-tdaB | 42 | 0.0 | 0.0 | 84.0 | -2.6 | 0.036 | ***Roseitalea* ASV47** |
| Control - WT | 42 | 0.0 | 0.0 | 85.2 | -2.8 | 0.017 | ***Roseitalea* ASV47** |
| d-tdaB - WT | 42 | 0.0 | 0.0 | 85.2 | -0.5 | 1.000 | ***Roseitalea* ASV47** |
| Control - d-tdaB | 56 | 0.0 | 0.0 | 84.0 | -3.3 | 0.005 | ***Roseitalea* ASV47** |
| Control - WT | 56 | 0.0 | 0.0 | 86.4 | -3.0 | 0.011 | ***Roseitalea* ASV47** |
| d-tdaB - WT | 56 | 0.0 | 0.0 | 86.4 | -0.3 | 1.000 | ***Roseitalea* ASV47** |

| **Table S5.** TDA-producing *P. inhibens* (WT) has no major genetic adaption to the microalgal environment. Mutations in 12 ancestral (day 0) and 36 evolved (day 35, 63 and 70) *P. inhibens* (WT) isolates, isolated from the *Tetraselmis suecica* systems exposed to *P. inhiben*s (WT) at day 0. Only unique mutations are shown. In addition, all 48 isolates had two insertions at position 37 and 153 514 ( G → GT and G → GC, respectively). | | | | | | |
| --- | --- | --- | --- | --- | --- | --- |
|  |  |  |  |  |  |  |
| **Isolate** | **P2_T9_1** | **P3_T10_4** | **P3_T10_9** | **P4_T0_1** | **P4_T0_4** | **P4_T10_8** |
| **Origin (lineage)** | 2 | 3 | 3 | 4 | 4 | 4 |
| **Day** | 63 | 70 | 70 | 0 | 0 | 70 |
| **Chromosome** | CP002976 | CP002976 | CP002976 | CP002977 | CP002976 | CP002976 |
| **Position** | 1962876 | 1706383 | 2504725 | 87677 | 2098790 | 673650 |
| **Type** | snp | snp | snp | ins | snp | snp |
| **Reference** | C | C | G | A | G | T |
| **Alteration** | T | A | C | AC | C | G |
| **Evidence** | T:250 C:0 | A:188 C:1 | C:261 G:0 | AC:241 A:0 | C:204 G:0 | G:250 T:0 |
| **Ftype** | CDS | CDS |  | CDS | CDS | CDS |
| **Strand** | - | - |  | - | + | + |
| **NT position** | 1635/1662 | 6/780 |  | 762/1110 | 1732/1752 | 617/1347 |
| **AA Position** | 545/553 | 2/259 |  | 254/369 | 578/583 | 206/448 |
| **Effect** | synonymous_variant c.1635G>A p.Arg545Arg | synonymous_variant c.6G>T p.Thr2Thr |  | frameshift_variant c.762dupG p.Ser255fs | missense_variant c.1732G>C p.Gly578Arg | missense_variant c.617T>G p.Val206Gly |
| **Locus tag** | PGA1_c18920 | PGA1_c16450 |  | PGA1_262p00780 | PGA1_c20210 | PGA1_c06550 |
| **Gene** | secD |  |  |  | ilvI2 | glmM |
| **Product** | protein-export membrane protein SecD | hypothetical protein |  | NAD dependent epimerase/dehydratase | acetolactate synthase isozyme 3 large subunit | phosphoglucosamine mutase GlmM |
